# Targeting the Chemokine System in Rheumatoid Arthritis and Vasculitis

**DOI:** 10.31662/jmaj.2020-0019

**Published:** 2020-07-13

**Authors:** Yoshishige Miyabe, Chie Miyabe, Yoshiko Iwai, Andrew D. Luster

**Affiliations:** 1 Department of Cell Biology, Institute for Advanced Medical Sciences, Nippon Medical School, Tokyo, Japan; 2 Department of Dermatology, Tohoku Medical and Pharmaceutical University, Sendai, Japan; 3 Center for Immunology and Inflammatory Diseases, Division of Rheumatology, Allergy and Immunology, Massachusetts General Hospital, Harvard Medical School, Boston, USA

**Keywords:** Chemokine, Rheumatoid arthritis, Vasculitis

## Abstract

Arrest of circulating leukocytes and subsequent diapedesis is a fundamental component of inflammation. In general, the leukocyte migration cascade is tightly regulated by chemoattractants, such as chemokines. Chemokines, small secreted chemotactic cytokines, as well as their G-protein-coupled seven transmembrane spanning receptors, control the migratory patterns, positioning and cellular interactions of immune cells. Increased levels of chemokines and their receptors are found in the blood and within inflamed tissue in patients with rheumatoid arthritis (RA) and vasculitis. Chemokine ligand-receptor interactions regulate the recruitment of leukocytes into tissue, thus contributing in important ways to the pathogenesis of RA and vasculitis. Despite the fact that blockade of chemokines and chemokine receptors in animal models have yielded promising results, human clinical trials in RA using inhibitors of chemokines and their receptors have generally failed to show clinical benefits. However, recent early phase clinical trials suggest that strategies blocking specific chemokines may have clinical benefits in RA, demonstrating that the chemokine system remains a promising therapeutic target for rheumatic diseases, such as RA and vasuculitis and requires further study.

## 1. Introduction

Egress of leukocytes from the circulation into the extravascular space is a fundamental component of inflammation. Leukocyte recruitment from blood into tissues follows a well-established paradigm: (i) tethering and rolling on the vessel wall, (ii) firm arrest on the endothelium, (iii) spreading out and crawling in all directions on the vessel, and transendothelial migration to extravasate into tissue. This process is tightly controlled by adhesion molecules and chemoattractants including chemokines ^(1)^.

Chemokines are chemotactic cytokines that regulate the migration of a wide variety of immune cells ^(2)^. In the context of the immune system, chemokines control the positioning of all immune cells and contribute to immune cell development, homeostasis and inflammation. More than 50 chemokines and 19 chemokine receptors have been identified in humans and mice (Table 1) ^(2)^. These small (8-10 kDa) secreted proteins are classified into four groups on the location of cysteine residues near the N terminus of their primary amino acid sequence: XC-chemokines contain a single N-terminal cysteine, CC-chemokines have two adjacent residues, CXC-chemokines have two cysteines separated by one other amino acid, and CX_3_C-chemokines have three amino acids between the cysteine residues ^(2)^. Most chemokines are secreted into the extracellular space where they bind heparin-like glycosaminoglycans on the cell surface and are embedded in the extracellular matrix, forming transient or stable gradients. Chemokines and their gradients are detected by binding to specific chemokine receptors. Chemokine receptors are seven-transmembrane receptors and are expressed on the surface of immune cells. These receptors are G-protein coupled receptors that signal via Gi-type G proteins. There are approximately 20 *classical* chemokine receptors coupled to G proteins and regulate cell migration, whereas 5 *atypical* chemokine receptors that do not couple to G proteins and do not induce cell migration have been identified (Table 2) ^(2)^.

**Table 1.** Chemokines and Their Receptors.

Chemokine	Other names	Receptor
**CXCL1**	GROα, MGSA, mouse KC	CXCR2, ACKR1
**CXCL2**	GROβ, MIP-2α, mouse MIP2	CXCR2, ACKR1
**CXCL3**	GROγ, MIP-2β	CXCR2, ACKR1
**CXCL4**	PF4	Unknown
**CXCL4L1**	PF4V1	Unknown
**CXCL5**	ENA-78, mouse LIX^a^	CXCR2, ACKR1
**CXCL6**	GCP-2 (human only)	CXCR1, CXCR2, ACKR1
**CXCL7**	NAP-2	CXCR2, ACKR1
**CXCL8**	IL-8 (human only)	CXCR1, CXCR2, ACKR1
**CXCL9**	Mig	CXCR3
**CXCL10**	IP-10	CXCR3
**CXCL11**	I-TAC	CXCR3, ACKR1, ACKR3
**CXCL12**	SDF-1	CXCR4, ACKR3
**CXCL13**	BLC, BCA-1	CXCR5, ACKR1, ACKR4
**CXCL14**	BRAK	Unknown
**CXCL15**	Lungkine (mouse only)	Unknown
**CXCL16**		CXCR6
**CCL1**	I-309, mouse TCA3	CCR8
**CCL2**	MCP-1, mouse JE	CCR2, ACKR1, ACKR2
**CCL3^b^**	MIP-1α, LD78α	CCR1, CCR5, ACKR2
**CCL3L1**	LD78β	CCR1, CCR3, CCR5, ACKR2
**CCL4**	MIP-1β	CCR5, ACKR2
**CCL4L1**	LAG-1	CCR5
**CCL5**	RANTES	CCR1, CCR3, CCR5, ACKR2
**CCL6**	C-10, MRP-1 (mouse only)	Unknown
**CCL7**	MCP-3, mouse Fic or MARC	CCR2, CCR3, ACKR1, ACKR2
**CCL8 **	MCP-2	Human: CCR1, CCR2, CCR3, CCR5, ACKR1, ACKR2; mouse: CCR8, ACKR1, ACKR2
**CCL9/10**	MIP-1γ, MRP-2 (mouse only)	Unknown
**CCL11**	Eotaxin-1	CCR3, ACKR2
**CCL12**	MCP-5 (mouse only)	CCR2
**CCL13**	MCP-4 (human only)	CCR2, CCR3, CCR5, ACKR1, ACKR2
**CCL14**	HCC-1 (human only)	CCR1, ACKR1, ACKR2
**CCL15**	Leukotactin-1, HCC-2, MIP-5 (human only)	CCR1, CCR3
**CCL16**	HCC-4, NCC-4, LEC (human only)	CCR1, CCR2, CCR5, ACKR1
**CCL17**	TARC	CCR4, ACKR1, ACKR2
**CCL18**	PARC, DC-CK1 (human only)	CCR8
**CCL19**	MIP-3β, ELC	CCR7, ACKR4
**CCL20**	MIP-3α, LARC	CCR6
**CCL21**	SLC, 6CKine	CCR6, CCR7, ACKR4
**CCL22**	MDC	CCR4, ACKR1, ACKR2
**CCL23**	MPIF-1, MMP-3 (human only)	Unknown
**CCL24**	Eotaxin-2, MPIF-2	CCR3
**CCL25**	TECK	CCR9, ACKR4
**CCL26**	Eotaxin-3	CCR3, CX_3_CR1
**CCL27**	CTAK	CCR10
**CCL28**	MEC	CCR3, CCR10
**XCL1**	Lymphotactin α, SCM-1α	XCR1
**XCL2 **	Lymphotactin β, SCM-1β	XCR1
**CX_3_CL1**	Fractalkine	CX_3_CR1

**Table 2.** Chemokine Receptors: Cellular Expression and Primary Function.

Receptor	Cell expression	General immune function
Classical chemokine receptors		
CXCR1	Neutrophils and lower expression on monocytes, NK cells, mast cells, basophils, CD8^+^ T_EFF_ cells and endothelial cells	Neutrophil trafficking
CXCR2	Neutrophils and lower expression on monocytes, NK cells, mast cells, basophils, CD8^+ ^T cells and endothelial cells	B cell lymphopoiesis, neutrophil egress from bone marrow and neutrophil trafficking
CXCR3	T_H_1 cells, CD8^+^T_CM_ cells and T_EM _cells, NK cells, NKT cells, plasmacytoid dendritic cells, B cells, T_reg _cells and T_FH _cells	Type 1 adaptive immunity
CXCR4	Most leukocytes and endothelial cells	Hematopoiesis, organogenesis and bone marrow homing
CXCR5	B cells, T_FH_ cells, T_FR_ cells CD8^+^ T_EM_ cells	B cell and T cell trafficking in lymphoid tissue to the B cell zone or follicles
CXCR6	T_H_1 cells, T_H_17 cells, γδT cells, ILCs, NKT cells, NK cells, plasma cells, endothelial cells	ILC function and adaptive immunity
CCR1	Monocytes, macrophages, neutrophils, T_H_1 cells, basophils, dendritic cells	Innate immunity and adaptive immunity
CCR2	Monocytes, macrophages, T_H_1 cells, immature dendritic cells, basophils, NK cells and endothelial cells	Monocyte trafficking and type 1 adaptive immunity
CCR3	Eosinophils and lower expression on basophils and mast cells	Type 2 adaptive immunity, eosinophil distribution and trafficking
CCR4	T_H_2 cells, skin-homing T cells and lung-homing T cells, T_reg _cells and lower expression on T_H_17 cells, CD8^+^T cells, monocytes, B cells and immature dendritic cells	Homing of T cells to the skin and lungs and type 2 immune responses
CCR5	Monocytes, macrophages, T_H_1 cells, NK cells, T_reg_ cells, CD8^+ ^T cells, dendritic cells and neutrophils	Type 1 adaptive immunity
CCR6	T_H_17 cells, immature dendritic cells, γδT cells, NKT cells, NK cells, T_reg_ cells and T_FH _cells	Immature dendritic cell trafficking, GALT development and type 17 adaptive immune responses
CCR7	Naïve T cells, T_CM _cells, T_RCM _cells, mature dendritic, B cells	Mature dendritic cell, B cell and T cell trafficking in lymphoid tissue to T cell zone and egress of dendritic cells and T cells from tissue
CCR8	T_H_2 cells, T_reg _cells, skin T_RM _cells, γδT cells, monocytes and macrophage	Immune surveillance in skin, type 2 adaptive immunity and thymopoiesis
CCR9	Gut-homing T cells, thymocytes, B cells, dendritic cells and plasmacytoid dendritic cells	Homing of T cells to the gut, GALT development and function and thymopoiesis
CCR10	Skin-homing T cells and IgA-plasmablasts	Homing immunity at mucosal sites and immune surveillance in the skin
XCR1	Cross-presenting CD8^+^ dendritic cells and thymic dendritic cells	Antigen cross-presentation by CD8^+^ dendritic cells
CX_3_CR1	Resident monocytes, macrophages, microglia, T_H_1 cells, CD8^+ ^T_EM _cells, NK cells, γδT cells and dendritic cells	Patrolling monocytes in innate immunity, microglial cell and NK cell migration and type 1 adaptive immunity
Atypical chemokine receptors		
ACKR1 (DARC)	Red blood cells and endothelial cells	Chemokine transcytosis and chemokine scavenging
ACKR2 (D6)	Dendritic cells, B cells and lymphatic endothelium	Chemokine scavenging
ACKR3 (CXCR7)	B cell and stromal cells	Shaping chemokine gradients for CXCR4
ACKR4 (CCRL1; CCX-CKR)	Thymic epithelium	Chemokine scavenging

DARC, Duffy Antigen Receptor for Chemokines; GALT, gut-associated lymphoid tissue; ILCs, innate lymphoid cells; NK, natural killer; NKT, natural killer T; T_CM _cell, central memory T cell; T_EFF _cell, effector T cell; T_EM _cell, effector-memory T cell; T_FH _cell, follicular helper T cell; T_FR _cell, follicular regulatory T cell; T_H _cell, T helper; T_RCM_, recirculating memory T cell; T_REG _cell, regulatory T cell; T_RM _cell, resident-memory T cell.

The chemokine system and other inflammatory mediators might play a central role in chronic inflammation such as autoimmune diseases. In fact, high levels of several chemokines are seen in the blood and disease tissues, such as the joints and blood vessels, in patients with rheumatoid arthritis (RA) ^(3)^ and vasculitis ^(4)^, and their levels have been associated with the disease severity and/or activity. Chemokines and their receptors likely contribute to the recruitment of immune cells into the affected tissue in RA and vasculitis and are also thought to contribute to the activation of leukocytes once in the tissue, resulting in integrin activation and the production of proteases and inflammatory mediators ^(5)^. Thus, the control of leukocyte entry into the tissue represents a major point at which new therapeutics, such as blockade of the chemokine system, could be developed to attenuate inflammation in RA and vasculitis. 

In this review, we summarize the pathogenic roles of chemokines and their receptors in RA and vasculitis. Additionally, we provide an update on the clinical trials of chemokine- and chemokine-receptor-targeting drugs in arthritis and vasculitis and discuss their potential as therapeutic targets.

## 2. Rheumatoid Arthritis

Inflammatory arthritis, including rheumatoid arthritis (RA), is characterized by immune cell infiltration into joints, resulting in pannus formation with associated cartilage and bone destruction. This immune cell infiltrate drives RA pathogenesis and is composed of multiple cell types, including lymphocytes, macrophages, and neutrophils. Approximately 1 million people in Japan have RA. If not effectively treated, RA causes irreversible joint destruction resulting in significant disability, increased risk of cardiovascular disease, and markedly increased medical costs. Biological therapies, such as TNF inhibitors and IL-6 inhibitors, have revolutionized the treatment of RA. However, ~50% of RA patients still do not respond to these agents, and patients who do respond often have a residual disease and are at an increased risk of infection, such as pneumonia, associated with their use ^(6)^. Therefore, there remains an ongoing need to identify new therapeutic targets and treatment strategies for RA.

Animal models of inflammatory arthritis have provided valuable research tools for understanding the pathogenic mechanism of RA and for studying therapeutic targets. In fact, several animal models of inflammatory arthritis, such as the K/BxN arthritogenic serum transfer model of arthritis ^(7)^, the type II collagen-induced arthritis (CIA) ^(8)^ and the collagen antibody-induced arthritis (CAIA) ^(9)^, have a number of similarities to RA, including synovial hyperplasia, mononuclear cell infiltration and bone destruction. Therefore, these animal models have been used to define the role of chemokines and their receptors in inflammatory arthritis. In this section, we describe the functional role of chemokines and their receptors in animal models and in human RA and discuss therapies targeting these molecules.

### 2.1 Chemokine in RA

Numerous studies have demonstrated that multiple chemokines are highly expressed in serum, synovial fluid and synovial tissue of patients with RA, compared to healthy controls. For instance, the CXC-chemokines (CXCL5, CXCL8, CXCL9, CXCL10, CXCL12, CXCL13 and CXCL16), the CC-chemokines (CCL2, CCL3, CCL4, CCL5, CCL18, CCL19, CCL20, CCL21, CCL25), and the CX_3_C-chemokine CX_3_CL1 are increased in serum, synovial fluid, and synovial tissue of RA patients, compared with healthy controls ^(10), (11), (12), (13), (14), (15)^. In the pathogenesis of human RA, CXC-chemokines promote neutrophils (CXCL1, CXCL2, CXCL5 and CXCL8) ^(16)^, the effector T cell (CXCL10) ^(17)^ and the B cell (CXCL13) ^(18), (19)^ recruitment into the joint. CC-chemokines induce monocytes (CCL2, CCL3, CCL4, CCL5 and CCL7) ^(20), (21)^, T cells (CCL18, CCL19, CCL20, CCL21 and CCL25) ^(22)^ and the B cell (CCL20) ^(23)^ entry into the inflamed joint, whereas CX_3_CL1 induces monocyte recruitment ^(24)^.

In addition, CXCL8, CXCL12 and CXCL16 induce angiogenesis in synovial tissue in RA, and CXCL8, CXCL10 and CXCL13 are promising biomarkers of RA disease activity/severity ^(11), (25)^. Synovial macrophages and fibroblast-like synoviocytes (FLSs) might be the main producers of many inflammatory chemokines in the joints of RA patients, whereas synovial endothelial cells mainly produce CX_3_CL1 and some CC-chemokines in RA patients. However, the pathogenic roles of chemokines in human RA remain unclear.

**Figure 1.**
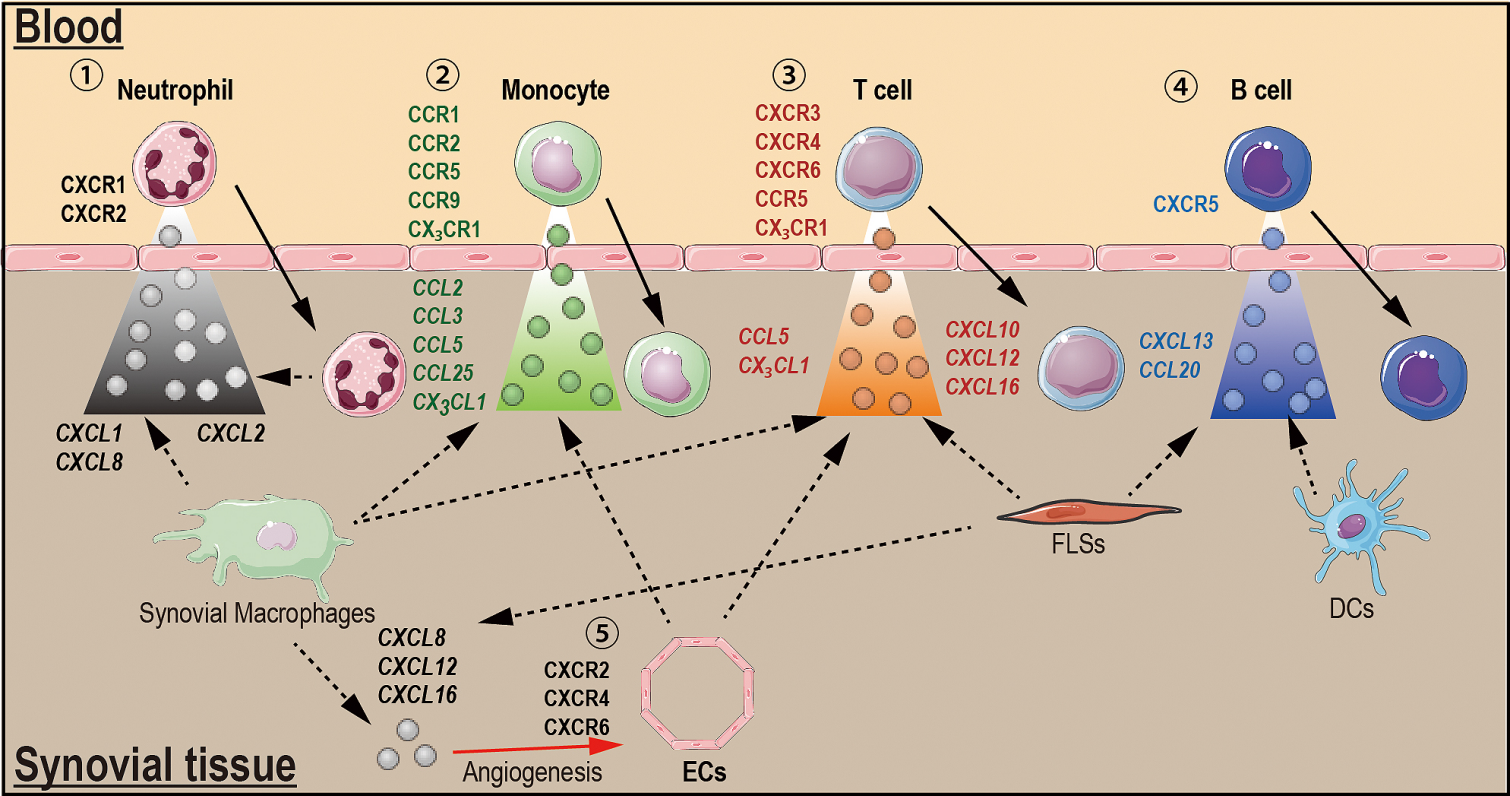
Chemokines and chemokine receptors in rheumatoid arthritis. ①Neutrophil recruitment: Synovial macrophages generate CXCL1 and CXCL8, which promote neutrophil recruitment into the joint. Once neutrophils enter into the joint, they become activated and produce CXCL2, which amplifies neutrophil recruitment and stimulates additional chemokine production by macrophages. ②Monocyte recruitment: Synovial macrophages generate chemokines that promote monocyte recruitment through CCR2, CCR5, CCR9 and CX_3_CR1. ③T cell recruitment: Synovial macrophages, endothelial cells (ECs) and fibroblast-like synovial cells (FLSs) generate chemokines that induce T cell trafficking through CXCR3, CXCR4, CXCR6, CCR5 and CXCR1. ④B cell recruitment; FLSs and dendritic cells (DCs) produce CXCL13 and CCL20 that promote B cell recruitment through CXCR5. ⑤Angiogenesis: CXCL8, CXCL12 and CXCL16 produced by synovial macrophage and FLSs induce angiogenesis through CXCR2, CXCR4, and CXCR6, respectively, expressed on ECs. Solid arrows depict cell movement in response to chemokines, and dashed arrows indicate chemokine secretion from cell. The red solid arrow indicates chemokine-inducing angiogenesis.

These same chemokines are also elevated in the serum, synovial fluid and synovial tissue in animal models of inflammatory arthritis, compared with control animals ^(20), (21), (22), (24), (26), (27), (28), (29), (30), (31), (32)^. Thus, animal models of arthritis are available to dissect the pathogenic roles of chemokines in inflammatory arthritis. Several studies using the animal models have indicated the development of arthritis in RA: (i) immune complexes stimulate synovial macrophages to generate CXCL1 that initiates neutrophil recruitment into the joints in the early phase of animal models of arthritis ^(33)^; (ii) after neutrophils enter the inflamed tissue, they become activated and produce CXCL2, which contributes to the recruitment of additional neutrophils in a positive-feedback loop ^(26), (33)^; (iii) neutrophils can also amplify the local response by secreting inflammatory cytokines, such as IL-1, that induce chemokine production by FLSs in the joint ^(26)^; (iv) In later phases of the disease, FLSs and other cells might play important roles in recruiting T and B cells (Figure 1).

### 2.2 Chemokine receptors in RA

Multiple chemokine receptors (CXCR1-6, CCR1, CCR2, CCR5-7, CCR9 and CCR10) and chemokines are all highly expressed in the joints of human RA and in animal models of arthritis ^(10), (20), (22), (26), (27), (34), (35)^. In addition, chemokine receptors control the recruitment of neutrophils (CXCR1 and CXCR2), T helper type 1 (T_H_1) cell (CXCR3), lymphocyte (CXCR4), B cell (CXCR5), T_FH_ cells (CXCR5), T cell (CXCR6, CCR5 and CCR6), monocyte (CCR1, CCR2, CCR5-7, CCR9 and CCR10) and DCs (CCR9) into the inflamed joints in human RA and in animal models (Figure 1) ^(10), (17), (18), (19), (20), (22), (23), (26), (35), (36)^. The CX_3_C-chemokine receptor CX_3_CR1 is expressed on CD3-positive T cells in the RA synovium ^(15)^, suggesting that the receptor also contributes to the recruitment of T cells into RA joints (Figure 1). Thus, different combinations of chemokine receptors are required for the entry of different cell types into the synovium during RA. For instance, CXCR1 and CXCR2 are required for neutrophils, CCR2, CCR5, CCR9, and CX_3_CR1 for monocytes; CXCR3, CXCR4, CXCR6, CCR5, CCR6 and CX_3_CR1 for T cells; and CXCR5 for B cells (Figure 1).

Some chemokine receptors are also expressed on stromal cells in RA patients, although their functions on these cells are largely unknown. Although chemokine receptor signaling in synovial endothelial cells (ECs) during RA may be an important mediator of angiogenesis, others in RA FLSs may contribute to induce the production of inflammatory mediators. For instance, CCL21/CCR7 and CCL28/CCR10 signaling in synovial ECs promotes RA angiogenesis ^(37), (38)^. CCR9, which is highly expressed on FLSs during RA, induces the production of inflammatory cytokines *in vitro *upon stimulation with CCL25, although the role of this interaction *in vivo* is not yet clear ^(22)^. These studies suggest that in addition to immune cells, the chemokine system also plays important roles in the function of stromal cells, such as ECs and FLSs, in the pathogenesis of RA.

*Atypical* chemokine receptors (ACKRs) are homologous to *classical* chemokine receptors and bind chemokines but do not couple to G proteins. These atypical receptors appear to play a primary role in shaping chemokine gradients by scavenging and controlling transcytosis of chemokines. Atypical chemokine receptor 1 (ACKR1/DARC) and ACKR3/CXCR7 are expressed on joint ECs in human RA synovial tissue ^(13), (39), (40)^. It has recently been reported that ACKR2/D6 expression is elevated on peripheral blood cells and on leukocytes and stromal cells in synovial tissue of RA patients ^(41)^. More recently, we have demonstrated that ACKR1/DARC has transcytosed inflammatory chemokines (CXCL1 and CXCL2) to initiate neutrophil entry into the joint in animal models of inflammatory arthritis ^(39), (42)^. Meanwhile, ACKR3/CXCR7 has been demonstrated to play a role in angiogenesis in an animal model of arthritis ^(13)^. However, the functional role of ACKRs in human RA pathogenesis remains unknown.

### 2.3 Targeting the chemokine system in RA

Numerous chemokines and their receptor inhibitors have been investigated and tested in the animal models. For instance, blockade of a single chemokine (CXCL10, CXCL13, CCL2, CX_3_CL1 or XCL1) or chemokine receptors (CXCR2, CXCR3, CXCR5, CXCR7, CCR1, CCR2, CCR5, CCR7 and CCR9) have demonstrated preventive and, in some cases, therapeutic efficacy in animal models ^(13), (17), (21), (22), (24), (26), (27), (36), (43), (44), (45), (46), (47), (48), (49), (50), (51), (52), (53), (54)^. However, only a few clinical trials (CXCL10; MDX-10 ^(14)^, CX3CL1; E6011 and CCR1; CCX354-C ^(55)^) of 10 drugs that target the chemokine system had some clinical benefits in RA patients (Table 3).

**Table 3.** Targeting the Chemokine Systems in Rheumatoid Arthritis.

Target	Drug (Type of drug)	Type of study	Efficacy	Study outcome
CXCL10	MDX-1100 (antibody)	Phase II	Mildly effective	The ACR20 response at week 12 was 54% (MDX1100 and MTX) and 17% (placebo and MTX)
CCL2	ABN912 (antibody)	Phase Ib	Not effective	ABN912 did not result in any clinical improvement.
CCR1	CP-481,715 (small molecules)	Phase Ib	Mildly effective	CP-481,715 reduced tender and swollen joint count, and macrophages infiltration into the synovial tissue than those of placebo.
	CCX354-C (small molecules)	Phase II	Mildly effective	The ACR20 response at week 12 was 39% (placebo), 43% (CCX354-C; 100mg twice daily) and 52% (CCX354-C; 200 mg once daily)
	MLN3897 (small molecules)	Phase IIa	Not effective	The ACR20 response at week 12 was 35% (MLN3897) and 33% (placebo).
CCR2	MLN1202 (antibody)	Phase IIa	Not effective	Patients treated with CCR2 monoclonal antibody or placebo for 6 weeks. No clinical improvement
CCR5	SCH351125 (small molecules)	Phase Ib	Not effective	The ACR20 response at week 4 was 20% (SCH351125) and 33% (placebo).
	AZD5672 (small molecules)	Phase IIb	Not effective	The ACR20 response at week 12 was around 35% (AZD5672) and 38% (placebo).
	UK-427,857 (small molecules)	Phase IIa	Not effective	The ACR20 response at week 12 was 23.7% (UK-427,857) and 23.8% (placebo).
CX_3_CL1	E6011 (antibody)	Phase I/II	Effective? (no placebo)	~60% treated patients had at ACR20 response at week 12.

This table is modified from Miyabe Y, Lian J, Miyabe C, et al. Chemokines in rheumatic diseases: pathogenic role and therapeutic implications. Nat Rev Rheumatol. 2019;15(12):731-46 ^(5)^. The authors have the right to use the original table in Reference 5 and got the permission from Springer Nature.

Despite many inhibitors targeting the chemokine system failed to show positive results in clinical trials, indicating that the chemokine system might not be therapeutic targets for RA may be premature. CCR1 inhibitors might be an instructive example. MLN3897 was found to have a much lower pharmacokinetic and pharmacodynamic profile for CCR1 blockade compared to CCX354-C ^(56)^, suggesting that the lack of success of MLN3897 was not necessarily due to CCR1 being a poor target in terms of its underlying biology. Thus, CP-481,715 and MLN3897 failed to show any beneficial effects in clinical trials (Table 3) ^(55)^. It will be also important for inhibitors targeting the chemokine system to determine if optimum pharmacokinetic properties and receptor inhibition have been achieved before making final conclusions regarding the usefulness of inhibiting that particular target in a given disease process.

In addition, a more recent study of the animal model demonstrated that broadly cross-reactive chemokine-blocking antibodies for CXCR2 ligands (CXCL1-3 and CXCL5) dramatically attenuated inflammatory arthritis compared with blocking antibodies against a single chemokine (CXCL1) ^(57)^. Because the functions, such as adhesion and extravasation, during leukocyte trafficking overlaps between many chemokine systems, inhibition of a single chemokine system alone might not be sufficient to completely suppress leukocyte recruitment. Moreover, the pathogenesis of human RA is undoubtedly more complex than animal models of arthritis. Thus, strategies that inhibit *multiple* inflammatory chemokines, rather than a *single* chemokine alone, might also be a more promising direction for new RA therapies.

## 3. Vasculitis

Vasculitis is an autoimmune disease characterized by the presence of inflammatory leukocytes in vessel walls associated with destructive damage to vessel structures. The affected vessels vary in size, type, and location according to the type of vasculitic disease; (i) small vessels, e.g., ANCA-associated vasculitis (AAV); (ii) medium vessels, e.g., Kawasaki disease (KD); and (iii) large vessels, e.g., Takayasu disease (TD) and giant cell arteritis (GCA) ^(58)^. Corticosteroids and immunosuppressive agents, such as cyclophosphamide, methotrexate and tacrolimus, are commonly used for the treatment of vasculitis. However, in some cases, the disease is refractory to these treatments, and immunosuppression often leads to significant clinical complications. Therefore, there is a need for new therapies for vasculitis that are safer and more effective than currently used treatments.

Several experimental models of vasculitis have been developed, including *Polyoma* virus infection-induced vasculitis, ANCA-induced vasculitis and *Lactobacillus casei*-induced vasculitis, but the ability to induce vasculitis in these models is limited depending on the genetic background of the mouse strain ^(59)^. Injection of a *Candida albicans* water-soluble fraction (CAWS) into mice leads to inflammation of the aortic root and coronary arteries and can be used as a model for KD. CAWS-induced vasculitis can be induced in a variety of genetic backgrounds and is, therefore, more versatile compared to other animal models of studying the pathogenesis of vasculitis ^(59), (60)^. In CAWS-induced vasculitis, multiple immune cells such as neutrophils, macrophages and T cells, infiltrated the inflamed cites. Additionally, neutrophils might be a key driver of inflammation in CAWS-induced vasculitis ^(60)^.

### 3.1 Chemokines in vasculitis

Several chemokines are elevated in the plasma and serum of vasculitis patients and are correlated with disease activity (AAV: CCL17 ^(61)^, CCL18 ^(62)^, CCL20 ^(63)^, CXCL8-11 ^(4)^, CX_3_CL1 ^(64)^, and XCL1 ^(65)^; TD: CCL2 ^(66)^ and CCL5 ^(67)^; GCA: CCL2 ^(67)^, CXCL9-11 ^(68)^ and CX_3_CL1 ^(69)^; and KD: CCL17 ^(70)^ and CXCL9/10 ^(70), (71)^). In addition, CCL2, CCL7, CXCL2-3 and CXCL9-10 are highly expressed in the aortic root and coronary arteries of the CAWS-induced vasculitis mouse model ^(72), (73)^. Thus, the chemokine system also contributes to the development of vasculitis and RA.

**Figure 2.**
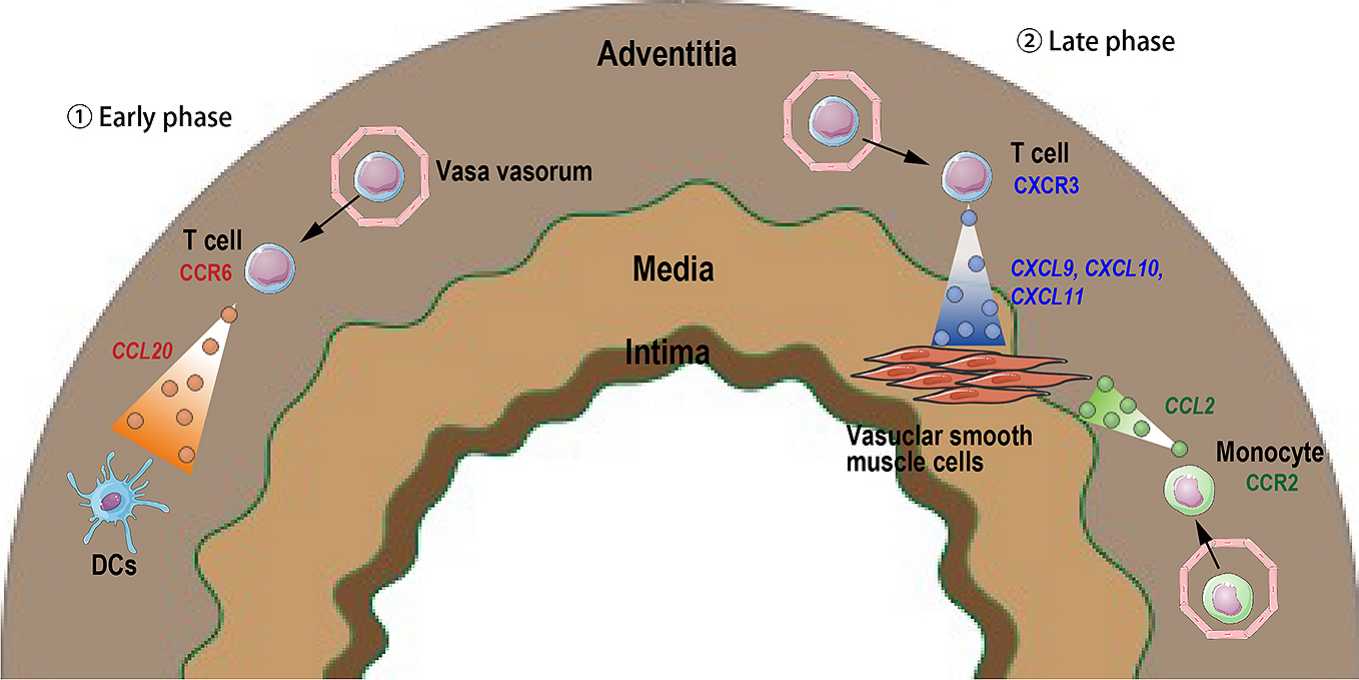
Chemokines and chemokine receptors in giant cell arteritis. ①Early phase: Tissue-resident dendritic cells (DCs) in adventitia produce CCL20 that promotes CCR6^+ ^T cell infiltration. ②Late phase: Vascular smooth muscle cells in media produce CXCL9, CXCL10 and CXCL11, which attract CXCR3^+ ^T cells. Vascular smooth muscle cells also produce CCL2 resulting in CCR2^+ ^monocyte recruitment. Solid arrows depict cell movement in response to chemokines, and dashed arrows indicate chemokine secretion from a cell.

In GCA, tissue-resident DCs in the adventitia of affected arteries may be a major chemokine producer in the early phase of disease onset, whereas vascular smooth muscle cells and inflammatory monocytes recruited into the artery generate chemokines during later phases (Figure 2) ^(74)^. More recently, we have demonstrated that Dectin-2-mediated CCL2 produced by tissue-resident macrophages in the aortic root and coronary arteries may be the key initiator of vascular inflammation in the CAWS-induced vasculitis. Thus, tissue-resident immune cells, such as tissue-resident macrophages and DCs, might be the main source of chemokines igniting vascular inflammation in the development of GCA and KD. However, the main sources and pathogenic role of chemokine system in other forms of human vasculitis and animal models are still unknown.

### 3.2 Chemokine receptors in vasculitis

CCR2 and CX_3_CR1 expression on monocytes and CXCR3 and CCR6 expression on CD8^+^ T cells, along with high levels of the ligands for these receptors, can be observed in temporal arterial biopsies derived from patients with active GCA (Figure 2) ^(68), (69)^. In addition, expression of CXCR2 and CCR2 in the aortic root and coronary arteries is increased in CAWS-induced vasculitis mice, suggesting that CXCR2 promotes neutrophil recruitment and CCR2 controls monocyte infiltration. However, the patterns of chemokine receptor expression and their pathogenic role in human and animal models of vasculitis have not been well studied yet.

### 3.3 Targeting the chemokine systems in vasculitis

CCR2-deficient mice are resistant to the development of CAWS-induced vasculitis, suggesting that chemokines and their receptors might be attractive new targets for vasculitis treatments ^(75)^. However, the effect of inhibiting chemokines and chemokine receptors in human vasculitis and in animal models has not been well studied yet. Recently, a complementary component C5a that is a chemoattractant, but not a chemokine, can be a new promising target for vasculitis. In fact, the C5a receptor inhibitor looks very promising in the clinical trial of ANCA-associated vasculitis ^(76)^. Thus, additional studies are required to establish a new vasculitis therapy for targeting the chemokine system.

## 4. Conclusion

Many chemokines and their receptors have been implicated in leukocyte recruitment into inflamed tissue in RA and vasculitis. Thus, chemokines and their receptors represent promising targets for therapeutic intervention in the treatment of RA and vasculitis. In fact, inhibition of chemokines and their receptors ameliorates inflammation in animal models. However, clinical trials with chemokine and chemokine receptor blockers in human RA have failed to show clinical effects. In addition, the mechanism of action for many chemokines and their receptors in RA and vasculitis is still unknown. An important observation that requires further understanding is that different chemokines may be important at different stages of disease depending on RA and vasculitis. Recent advances in imaging technology have provided unprecedented views into immune cell function in live animals, providing an entirely new paradigm for immune cell function. In fact, our recent work using multiphoton intravital microscopy to study neutrophil entry into the joint in the KxB/N model demonstrated that the *classical* C5a receptor C5aR1 and the *atypical* C5a receptor C5aR2 were required for initial integrin-dependent neutrophil arrest on the joint endothelium but were not involved in inducing neutrophil extravasation ^(16), (42)^. By contrast, the classical chemokine receptor CXCR2 and the atypical chemokine receptor ACKR1 were involved in neutrophil diapedesis into the joint space ^(16), (42)^. In addition, other work has indicated that blockade of multiple chemokines might be more effective than single chemokine inhibition, because functions of some chemokines in immune cell trafficking overlap ^(57)^. These studies point to a need to more fully dissect the functional roles of chemokines and their receptors in the pathogenesis of RA and vasculitis. A significant need remains for the treatment of RA and vasculitis. Recent clinical studies suggest that the development of more effective inhibitors of chemokines and their receptors has untapped therapeutic potential.

## Article Information

### 

This article is based on the study, which received the Medical Research Encouragement Prize of The Japan Medical Association in 2019.

### Conflicts of Interest

None

### Sources of Funding

This work of Y.M. was supported by the Japanese Society for the Promotion of Science (JSPS) Kakenhi grant number JP19K08895, AMED under Grant Number JPjm0210069h, the Takeda Science Foundation, the Maruyama Memorial Research Foundation, the NOVARTIS Foundation (Japan) for the Promotion of Science, and the Medical Research Encouragement Prize of The Japan Medical Association. The work of A.D.L. was supported by grants from the National Institutes of Health and the Rheumatology Research Foundation.
